# The First Complete Mitochondrial Genome of the Genus *Litostrophus*: Insights into the Rearrangement and Evolution of Mitochondrial Genomes in Diplopoda

**DOI:** 10.3390/genes15020254

**Published:** 2024-02-18

**Authors:** Gaoji Zhang, Ming Gao, Yukun Chen, Yinuo Wang, Tianyi Gan, Fuyuan Zhu, Hongyi Liu

**Affiliations:** 1 Co-Innovation Center for Sustainable Forestry in Southern China, Key Laboratory of State Forestry and Grassland Administration on Subtropical Forest Biodiversity Conservation, College of Life Sciences, Nanjing Forestry University, Nanjing 210037, China; zhanggaoji@njfu.edu.cn (G.Z.); 18934832876@163.com (M.G.); 2932797114@njfu.edu.cn (Y.C.); 17766100299@163.com (Y.W.); fyzhu@njfu.edu.cn (F.Z.); 2 College of Plant Health and Medicine, Qingdao Agricultural University, Qingdao 266109, China; gantianyi2004@163.com

**Keywords:** mitochondrial genome, Diplopoda, *L. scaber*, genomic features, phylogenetic analysis

## Abstract

This study presents the complete mitochondrial genome (mitogenome) of *Litostrophus scaber*, which is the first mitogenome of the genus *Litostrophus*. The mitogenome is a circular molecule with a length of 15,081 bp. The proportion of adenine and thymine (A + T) was 69.25%. The gene ND4L used TGA as the initiation codon, while the other PCGs utilized ATN (A, T, G, C) as the initiation codons. More than half of the PCGs used T as an incomplete termination codon. The transcription direction of the *L. scaber* mitogenome matched *Spirobolus bungii*, in contrast to most millipedes. Novel rearrangements were found in the *L. scaber* mitogenome: trnQ -trnC and trnL1- trnP underwent short-distance translocations and the gene block rrnS-rrnL-ND1 moved to a position between ND4 and ND5, resulting in the formation of a novel gene order. The phylogenetic analysis showed that *L. scaber* is most closely related to *S. bungii*, followed by *Narceus magnum*. These findings enhance our understanding of the rearrangement and evolution of Diplopoda mitogenomes.

## 1. Introduction

With more than 12,000 nominal species and almost 80,000 predicted species [1], the Diplopoda are a diverse group of arthropods. They are widely distributed [2]. Diplopoda are often colloquially called millipedes, but the majority possess only a few hundred pairs of legs or fewer [1]. As soil-based animals, millipedes are highly important components of terrestrial ecosystems [3], playing ecological roles in developed humus [3,4,5] and contributing to the decomposition of plant litter by breaking down fallen leaves and maintaining soil fertility [6]. Millipedes are usually used as bioindicators for soil quality. Due to their small size, as well as the diversity and abundance of arthropod groups, as bioindicators, they may more accurately reflect trends in species richness and community composition compared to vertebrates [1,2]. Therefore, many studies of the ecological functions of millipedes have been conducted [7,8,9]. Despite millipedes’ ubiquity, little research has been conducted regarding their evolutionary relationships. The classical taxonomy of millipedes is based on morphology and has defects; meanwhile, there is limited research on the population-level genetics of millipedes, leading to problems in the classification of this group [10,11]. Our understanding of millipedes’ higher-level relationships, ecology, behavior, physiology, and genomic composition is also limited.

The mitochondria are the basic organelle of eukaryotic cells, and they present independent and conserved genetic characteristic [12]. A typical animal mitochondria genome (mitogenome) is a circular structure with a length of 13–17 kbp. Mitogenomes usually comprise 37 coding genes, including 13 protein-coding genes (PCGs), 22 transfer RNA genes (tRNAs), 2 ribosomal RNA genes (rRNAs), and a D-loop region [13]. In arthropods, the mitogenome is more structurally diverse, and there are atypical mitogenomes. For example, the mitogenomes of certain species in the order Coleoptera are significantly larger than those of other studied arthropods [14]. Additionally, the mitogenomes of some species in the order Anoplura have been observed to split into minicircular chromosomes [15]. In recent years, mitogenomes have been widely used in studies of molecular systematics [16,17], population genetics [18,19], and molecular evolution [20,21]. In comparison with individual mitochondrial genes, which are susceptible to loss [22,23], mitogenomes provide greater accuracy when inferring phylogenetic relationships [24]. For example, there is evidence indicating that the mitochondrial cox1 gene is not suitable for making inferences about the phylogeny of many metazoans due to its low levels of polymorphism [25]. Among the characteristics of mitogenomes, gene rearrangements also provide insights into phylogenetic relationships [26]. The comparison of these gene arrangements has significant potential to help resolve some of the most fundamental branches of multicellular animal phylogeny [27]. Only recently have molecular phylogenetic techniques been used to reconstruct millipedes’ phylogenetic relationships. Myriapoda species are model organisms for studying the relationship between gene rearrangements and phylogenetic analysis due to their high rates of rearrangement [28]. Despite this potential, research on millipede mitogenomes remained limited [29,30]. At the time this paper was written, limited millipede mitogenomes have been uploaded to the NCBI website. However, some of these sequences may have annotation issues. To conduct a comprehensive and systematic phylogenetic analysis of the class Diplopoda, a large number of complete mitogenomes of its subordinate species are needed [31,32]. The lack of mitogenome data has led to insufficient research being conducted on the evolutionary significance of Diplopoda gene rearrangements and their phylogenetic relationships.

*Litostrophus scaber*, a common millipede species in China, belongs to the family Pachybolidae and the order Spirobolida. In this study, the mitogenome of *L. scaber* was assembled and characterized. We describe the genome size, nucleotide composition, codon usage, and gene rearrangements. To further investigate the use of complete or near-complete mitogenomes to enhance the effectiveness of phylogenetic analyses, we conducted phylogenetic analyses based on 13 PCGs to investigate the phylogenetic position of *L. scaber*. Our study aims to shed light on the diversity, evolution, gene rearrangement, and phylogenetic relationships of Diplopoda species.

## 2. Materials and Methods

### 2.1. Specimen Collection and DNA Extraction

The specimen used in this study was collected from the Seven Star Park (110.31° N, 25.27° E) in Guilin, Guangxi, China. After species diagnosis was performed based on morphological features given in previous research [2] and the distribution area provided by the Global Biodiversity Information Facility website (GBIF, available at https://www.gbif.org, accessed on 28 May 2023), the specimen was stored in a −80 °C refrigerator at Nanjing Forestry University Animal Molecular Evolution Laboratory. The collection of the specimen was reviewed and approved by Nanjing Forestry University. The specimen used in this study was collected and studied in accordance with Chinese laws. Total genomic DNA was extracted using a FastPure Cell/Tissue DNA Isolation Mini Kit (Vazyme, Nanjing, China), and it was then stored at −20 °C for the follow-up investigation.

### 2.2. Sequence Analysis

The complete genomic library of *L. scaber* was established using the Illumina platform (Personal, Shanghai, China), while the sequencing was performed using next-generation sequencing with an insert size of 300 bp (about 2 Gb of raw data). To generate clean data, low-quality sequences were removed. Then, 22,035,594 reads with a GC content of 31.69% were assembled to obtain a complete mitogenome using Geneious Prime v2023.1.2 software The mitogenome of *Spirobolus bungii* (Accession number: MT767838.1) was used as a template for the assembly. The medium sensitivity/speed option was used for the assembling. A consensus sequence was constructed using a 99% base call threshold.

The initial analysis of the mitogenome was based on DNASTAR Lasergene 7.1 and the MITOS Web Server (available at https://usegalaxy.eu/root?tool_id=toolshed.g2.bx.psu.edu%2Frepos%2Fiuc%2Fmitos%2Fmitos%2F1.1.1%20galaxy0, accessed on 6 June 2023) [33]. Lasergene 7.1 was used for the sequence alignment and gene recognition. The MITOS Web Server was utilized to locate RNA genes. The PCGs were predicted using both MITOS and the CD-search tool on the NCBI website (available at https://www.ncbi.nlm.nih.gov/, accessed on 9 June 2023). The correct mitogenome of *L. scaber* was submitted to GenBank (accession number: OR139892.1). The gene map was generated using the CG View Server (available at https://cgview.ca/, accessed on 17 June 2023). BLASTN was used for a mitogenomic comparison of 17 species and BRIG was used to acquire a graphical map of the BLASTN results [34]. The composition skew was calculated according to the following formula: AT-skew = (A − T)/(A + T) and GC-skew = (G − C)/(G + C) [35]. DNAsp was used to analyze nucleotide diversity (Pi) [36]. MEGA X was used to calculate the relative synonymous codon usage (RSCU) and non-synonymous (Ka) and synonymous substitutions (Ks). The ggplot2 and aplot packages, executed in R v.4.3.1, were utilized to generate these images. The secondary structures of 22 tRNA genes were predicted using ViennaRNA Web Services (available at http://rna.tbi.univie.ac.at/forna/, accessed on 23 June 2023) to generate colorful secondary structures [37].

### 2.3. Phylogenetic Analysis

A total of 23 mitogenomes with credible annotations were selected for phylogenetic analysis, representing 17 genera, 12 families, and 6 orders: the accession numbers and taxonomic information are presented in Table 1. A centipede species, *Cermatobius longicornis*, was used as the out-group. All operations were completed using the PhyloSuite v1.2.3 software package [38]. Sequences of 13 PCGs were extracted using the PhyloSuite v1.2.3 software package and were then used for the phylogenetic analyses. The ‘Normal’ or ‘Codon’ alignment and ‘auto’ strategy of MAFFT v7.313 were used for multiple gene alignment [39]. MACSE was used to optimize alignments using the classic “Needleman–Wunsch” algorithm [40]. Sequence pruning in the form of triplet codons was performed using the “Codon” pattern in Gblocks [41]. RNA sequences were aligned using the “automated1” mode in trimAI [42]. The constructed PCGs and RNA sequences generated concatenate sequences in PhyloSuite. With BIC as the standard criterion, partition analysis was performed for IQ-TREE and Mrbayes using ModelFinder’s Edge- unlinked mode. Each PCG was divided into three codon partitions using Codon Mode (3 sites) [43]. The best-fit models obtained using ModelFinder for both software packages are presented in Table S1. IQ-TREE was used to reconstruct the ML tree with 1000 bootstraps [44]. The BI tree was reconstructed using MrBayes 3.2.6 with four Markov chains (three hot chains and one cold chain). Two independent runs of 1000,000 generations were conducted, with sampling carried out every 1000 generations. The first 25% of samples were deleted to reduce the number of simulation errors [45]. Phylogenetic trees were visualized and edited using the Interactive Tree of Life Web Server (iTOL, available at https://itol.embl.de, accessed on 10 July 2023).

## 3. Results and Discussion

### 3.1. Mitogenomic Structure and Comparison

**Figure 1:**
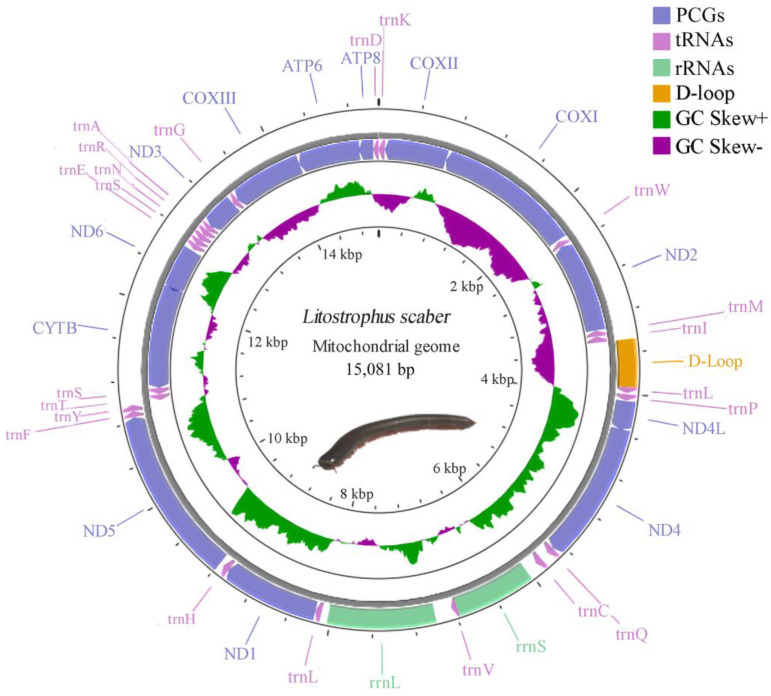
Circular map of the mitogenome of *Litostrophus scaber*. The outer ring represents genes encoded on the main stand (J-stand), and the inner ring represents genes encoded on the minor stand (N-stand). Genes are shown in different colors.

The mitogenome was a typical circular, double-stranded molecule; it was 15,081 bp in length, slightly longer than other species in the order Spirobolida. The mitogenome included 13 PCGs, 22 tRNAs, 2 rRNAs, and one D-loop region. Four PCGs (ND4L, ND4, ND1, and ND5), nine tRNAs (L1, P, Q, C, V, L2, H, F, and Y), and two rRNAs were translated on the majority stand (J-stand), which is similar to *S. bungii* in the same family [30] (Table 2, Figure 1). The base composition of the mitogenome was as follows: A 34.09%, T 35.16%, G 21.74%, and C 9.01%. An analysis of the base composition suggested that the whole mitogenome was biased toward A and T because of the high A + T content (69.25%). In addition, the AT skew was negative (−0.016) and the GC skew was positive (0.41). Two gene overlaps were identified, one between ND4L and ND4 (six bp in length) and the other between ATP6 and ATP8 (six bp in length).

**Figure 2:**
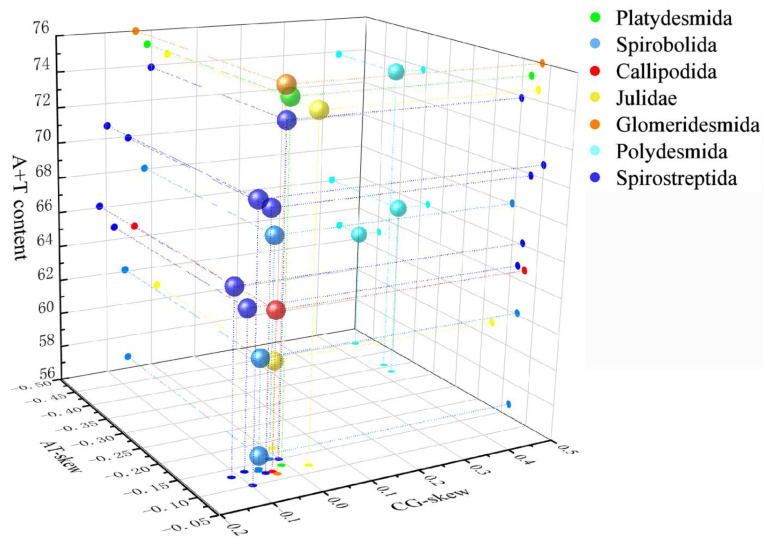
Three-dimensional scatter plots of the AT skew, GC skew, and A + T content of 16 Diplopod mitochondrial genomes. Balls of different colors correspond to different orders.

The A + T content, AT skew, and GC skew within Spirobolida exhibit significant variations (Figure 2). The A + T content ranges from 56.7% (*S. bungii*) to 68.04% (*L. scaber*). The AT skew ranges from −0.155 (*L. scaber*) to −0.131 (*N. annularus*), and the GC skew ranges from −0.060 (*N. annularus*) to −0.016 (*L. scaber*). The between-order analysis showed that the order Julida [46] and the order Polydesmida had T skews and G skews, while the other orders had the same skew as the order Spirobolida [47].

**Figure 3:**
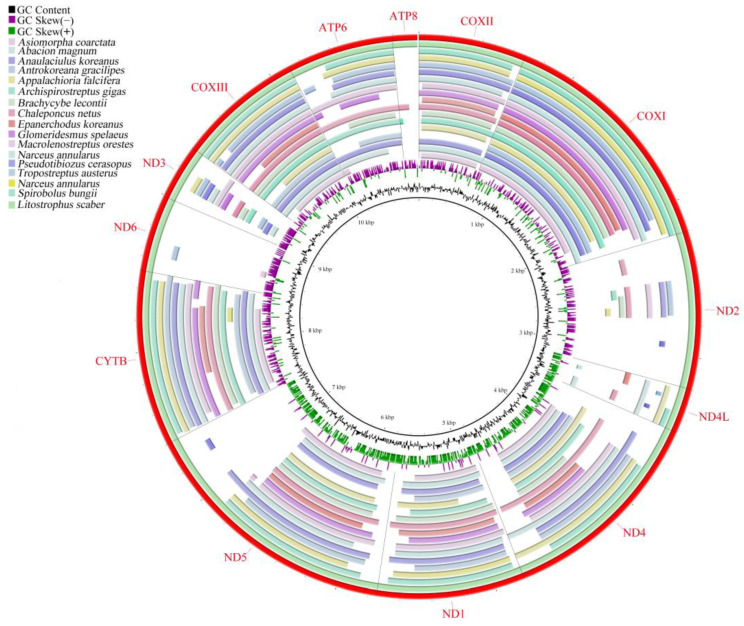
Graphical map of the BLAST results showing the nucleotide identity between the *Litostrophus scaber* mitochondrial genome and that of 16 other Diplopod species. Different colors correspond to different species.

The BLAST results revealed that *L. scaber* exhibited a high level of similarity to other millipedes at the protein level (Figure 3). The protein-level similarity ranged from 73.58% (*Appalachioria falcifera*) to 79.77% (*N. annularus*). Specifically, *L. scaber* exhibited the highest similarity with *N. annularus*, followed by *Chaleponcus netus* and *Prionopetalum kraepelini*. It is noteworthy that these similarities do not align consistently with the established phylogenetic relationships. This discrepancy might be attributed to the gene rearrangement, which appears to have a significant impact on the process of systematic evolution [48,49]. The charts generated from the BLAST results using BRIG indicated that the COX1 gene in millipedes was the most highly conserved, displaying the highest consistency. Moreover, the consistency of ATP8, ND6, ND2, and ND4L was lower, a result that aligns with findings from previous studies [30,31].

### 3.2. PCGs

**Figure 4:**
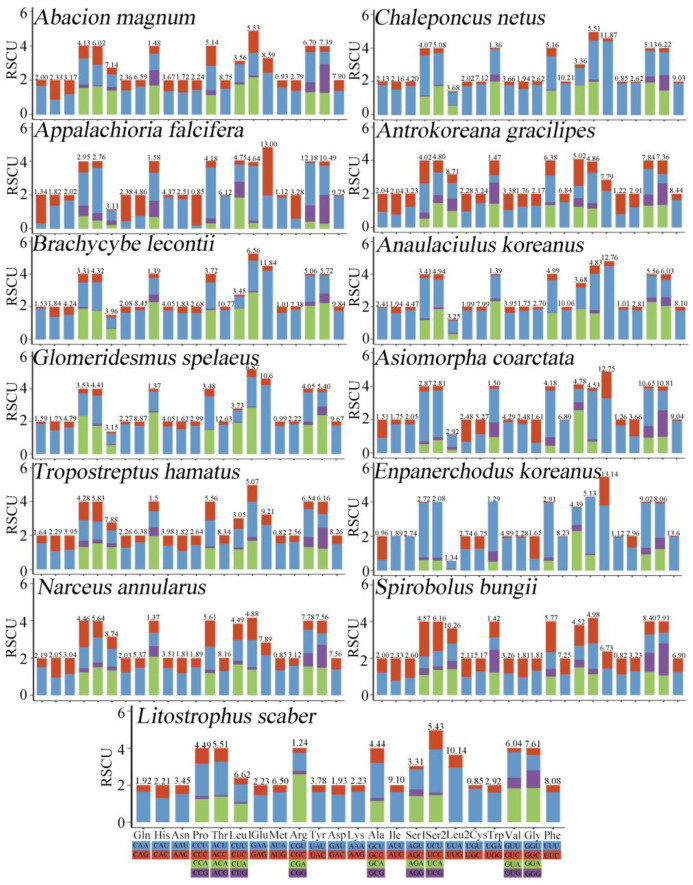
RSCU results of 13 species of Diplopoda, including the order Glomeridesmida, the order Julida, the order Spirobolida, the order Spirostreptida, the order Playtdesmida, and the order Polydesmida. Different colors correspond to a different third codon.

The total PCG length of the *L. scaber* mitogenome was 10,905 bp, accounting for 72.31% of the entire mitogenome, similar to other millipede species. Specifically, 12 PCGs used ATN (A, T, G, C) as the initiation codon, and the nonstandard initiation codon TGA was observed in ND4L. Unusual initiation codons have previously been reported, including ND1 of *Antrokoreana gracilipes*, which starts with GTG, and ATP8 of *Anaulaciulus koreanus*, which starts with TTA [46,47]. Seven PCGs, COX2, COX1, ND5, ND6, CYTB, COX3, and ATP6, used T as the termination codon. ND1 used TA as an incomplete termination codon. These special termination codons are also found in other arthropods [46,47], and these codons might be transformed into TAA or TAG for formal functions [50]. The remaining PCGs used TAA/TAG as the termination codons. The RSCU values of 13 millipede species from 12 families of 6 orders are summarized in Figure 4. Overall, the five amino acids with high usage levels were Leu, Gly, Val, Phe, and Ile. The four most common codons were UUU (Phe), AUU (Ile), UUA (Leu), and AUA (Met). The codons translated by these species ranged in quantity from 3628 to 3684. The usage of codons ending in A/U was significantly higher than that of codons ending in C/G, reflecting the strong AT bias of the third codon, a finding consistent with previous studies on the class Myriapoda [51,52]. Analogously, the biased use of A + T nucleotides was reflected in the codon frequencies.

**Figure 5:**
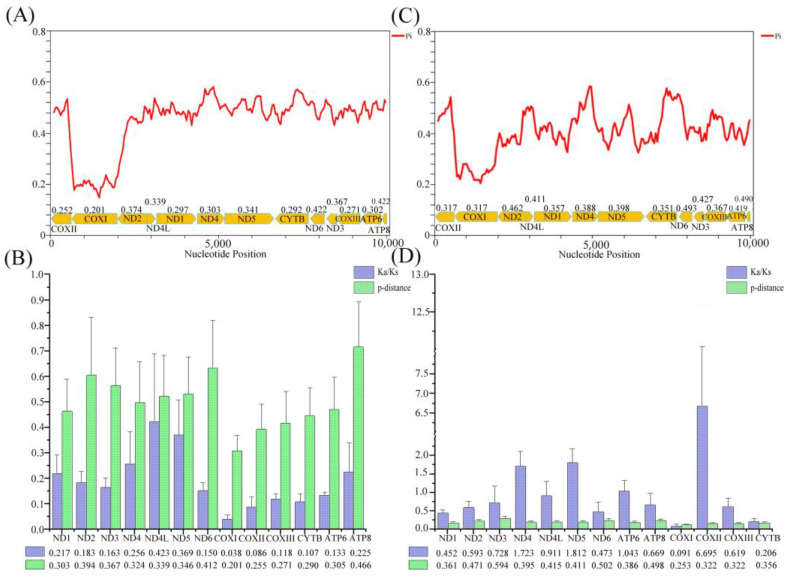
Variation in mitochondrial genes and the evolutionary characteristics of Diplopoda. (**A**) Sliding window analysis within the order Spirobolide, revealing the nucleotide diversity (Pi). (**B**) P-distance and Ka/Ks values of mitochondrial gene sequences within the order Spirobolide, revealing its evolutionary characteristics. (**C**) Sliding window analysis among Diplopoda, revealing Pi values. (**D**) P-distance and Ka/Ks values of mitochondrial gene sequences among Diplopoda, revealing their evolutionary characteristics.

Pi and P-distance analyses were performed to elucidate the variation and evolution of the PCGs (Figure 5). Within the order Spirobolida, sliding window analysis revealed variation in Pi among the PCGs. The average Pi of each gene ranged from 0.201 (COX1) to 0.422 (ATP8), with ATP8, ND6, and ND2 having the highest Pi values of 0.422, 0.422, and 0.374, respectively. Conversely, COX3, COX2, and COX1 had the lowest Pi values of 0.271, 0.252, and 0.201, respectively, in line with the genetic distance between the PCGs. This implies that ATP8, ND6, and ND2 are fast-evolving genes, while COX3, COX2, and COX1 are slow-evolving genes within the order Spirobolida. However, the analysis across different orders yielded different results. Specifically, within orders, ND3, ND6, and ATP8 were identified as fast-evolving genes, whereas COX3, COX2, and COX1 were determined to be slow-evolving genes in general. Overall, the results of our analysis were similar to those of previous studies [28,52].

To analyze the evolution rate of the PCGs, we assessed the average Ka/Ks values (Figure 5). Under the assumption of neutral protein-level evolution, the ratio of Ka to Ks should be equal, resulting in a Ka/Ks ratio of 1. A Ka/Ks ratio below 1 indicates the presence of purifying or stabilizing selection, which suggests a resistance to change. On the other hand, a ratio above 1 implies positive or Darwinian selection, which drives evolutionary change. Within the order Spirobolida, all PCGs had an average Ka/Ks value of less than 1, ranging from 0.038 (COX1) to 0.423 (ND4L), which indicated the presence of purifying selection. Moreover, among the orders, the average Ka/Ks values ranged from 0.206 (CYTB) to 6.695 (COX2), which suggested that ND4, ND5, ATP6, and COX2 had experienced positive selection. Specifically, COX2 exhibited a Ka/Ks value much higher than 1, this high value may be attributed to the limited number of species used for analysis. Different candidate markers can be selected based on different taxonomic categories. An analysis of the average Ka/Ks value between orders could potentially be used to select candidate markers for molecular biological identification across different orders.

### 3.3. RNA Genes and Non-Coding Regions

**Figure 6:**
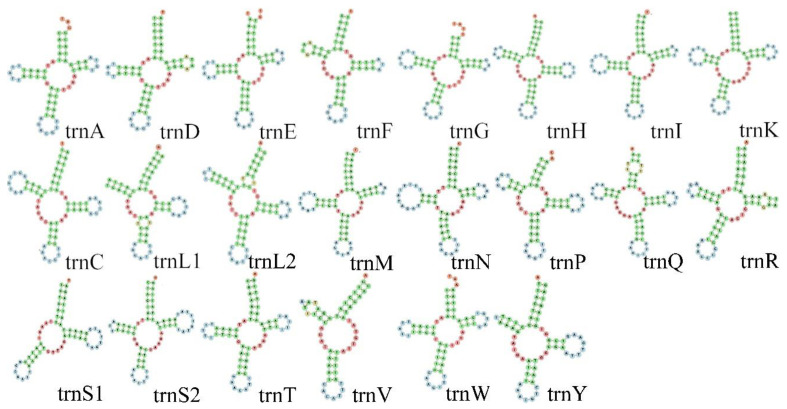
Secondary structure of 22 tRNA genes from the *Litostrophus scaber* mitochondrial genome.

The length of the tRNA genes ranged from 54 to 69 bp, and most tRNA’s arms formed classic Watson–Crick pairs. Moreover, 23 U-G wobble pairs that have specific functions were found in the tRNA [53], which is common in invertebrates [54] (Figure 6). The rrnS gene was located between trnV and trnC, with a length of 757 bp and a C + G content of 28.79%. The rrnL gene (length: 1031 bp) was located between trnV and trnL, with a C + G content of 28.42%. The rRNA region of the new mitogenomes was 1788 bp in length, accounting for 11.86% of the whole mitogenome.

The longest non-coding region (length: 459 bp) was flanked by trnI and trnL. This region is responsible for regulating transcription and replication. As the D-loop region of arthropods always has high A + T content [55], this area could be identified as the putative D-loop region based on its high A + T content (71.46%) compared to other mitochondrial genes of *L. scber*. There were also 17 intergenic regions in the mitogenome, which ranged in length from −6 to 169 bp, and the longest interval was found between rrnL and trnL. Specific spacers within these spacers may serve as binding sites for transcription termination factors [56].

### 3.4. Gene Rearrangement

**Figure 7:**
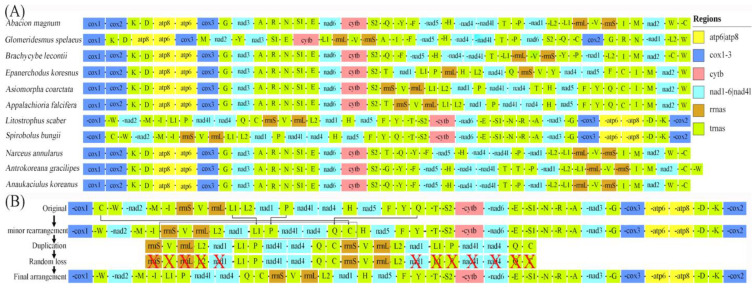
Comparison of mitogenome arrangements between different Diplopoda species. (**A**) Gene orders of the different types of mitogenome arrangement for the species used in this study. Gene segments are not drawn to scale. (**B**) The hypothetical process of the transposition of the gene block rrnS–rrnL–ND1 in the TDRL model. “X” indicates the partially random loss of the duplicated genes.

Comparisons of gene arrangements are an important tool for resolving deep phylogenetic relationships [57]. In millipedes, gene rearrangements occurred within and between orders (Figure 7). PCGs in *L. scaber* shared a similar transcription direction with *S. bungii*, in contrast to most millipedes [58]. Mitogenome arrangements vary significantly across the class Diplopoda. Gene rearrangements are observed, including both the RNA and PCGs. In terms of minor rearrangements, trnQ–trnC and trnL1–trnP underwent short-distance movements, resulting in the formation of trnQ–trnC–trnL1–trnP gene clusters. Rearrangements at the RNA level are common in arthropods [13,59], but these clusters are novel in millipedes. In terms of major rearrangements, the arrangement in *L. scaber* can be explained by a single rearranged gene block (rrnS–rrnL–ND1) moving to a position between ND4 and ND5 relative to *S. bungii* and *Narceus annulans*. The duplication–random loss (TDRL) model might be used to explain this arrangement [60]. According to this model, during replication, specific DNA segments are replicated at homologous sites and subsequently removed, resulting in the restoration of the original genomic organization or rearrangement [61]. To gain a deeper understanding of the evolutionary implications of gene arrangements in Diplopoda, it is necessary to conduct further research on mitochondrial genomes involving a wider range of taxa.

### 3.5. Phylogenetic Analysis

**Figure 8:**
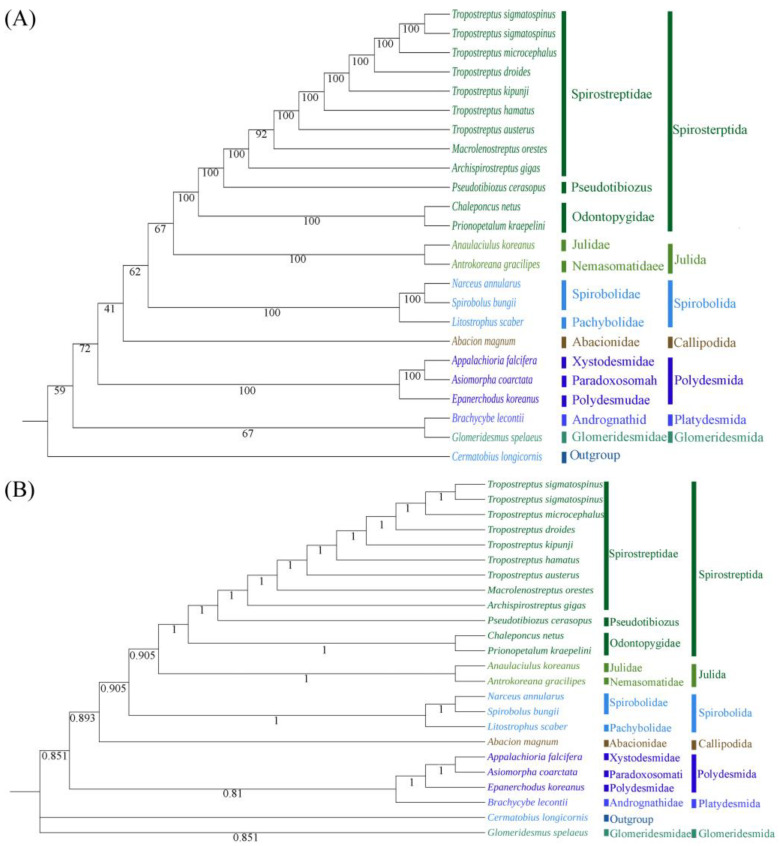
ML (**A**) and BI (**B**) trees based on the nucleotide datasets for 13 PCGs from the mitogenomes of 23 species. All the bootstrap values of the branches are indicated.

Because of the limited mitogenome sequences available for the class Diplopoda, we included only 22 millipede species in addition to the newly sequenced species from 17 genera of Diplopoda in the phylogenetic analyses; we selected *C. longicornis* as the out-group in the phylogenetic analysis of the class Diplopoda (Figure 8). The results from BI and ML exhibited striking similarities.

According to the current taxonomy, it is hypothesized that the orders Julida, Spirostreptida, and Spirobolida belong to the superorder Juliformia [62], but the specific relationships between these three orders remain controversial. A study based on mitogenomic analysis suggests that Julida and Spirostreptida have a sister-group relationship [63], while the morphological study suggested that Spirobolida and Julida have a sister-group relationship [60]. Studies based on mitochondrial genes, such as COX1 and rrnS, yield divergent perspectives [64,65]. The result obtained here were similar to those of previous phylogenetic studies based on the mitogenome. More mitogenomes will contribute to our understanding of the phylogenetic relationships between millipedes. Furthermore, our results showed a sister-group relationship between *N. annularus* and *S. bungii*, which did not agree with the result of Zuo Q. [28]. This might be due to the high levels of variation in the species selected for the two analyses.

## 4. Conclusions

In this study, we presented the newly sequenced mitogenome of *L. scaber*. This is the first representative mitogenome from the genus *Litostrophus*. The total length of the *L. scaber* mitogenome was 15,081 bp, with 69.25% A + T content. The mitogenome arrangement varies significantly in the class Diplopoda. Novel arrangements are found in the *L. scaber* mitogenome with the formation of trnQ–trnC and trnL–trnP gene clusters. The phylogenetic analysis indicated that *L. scaber* was a sister to *S. bungii*. The results obtained here were similar to those reported in previous phylogenetic studies based on the mitogenome. More mitogenomes will contribute to our understanding of the phylogenetic relationships between millipedes. Our study offers new insights into the evolution and phylogenetic relationships of Diplopoda.

## Figures and Tables

**Table 1:** List of complete mitogenomes used in this study.

Class	Order	Family	Genus	Species	Accession	Length (bp)
Diplopoda	Callipodida	Callipodidae	Abacion	*Abacion magnum*	JX437062.1	15,160
	Glomeridesmida	Glomeridesmidae	Glomeridesmus	*Glomeridesmus spelaeus*	MH590615.1	14,863
	Julida	Julidae	Anaulaciulus	*Anaulaciulus koreanus*	KX096886.1	14,916
		Nemasomatidae	Antrokoreana	*Antrokoreana gracilipes*	DQ344025.1	14,747
	Playtdesmida	Andrognathidae	Brachycybe	*Brachycybe lecontii*	JX437064.1	15,644
	Polydesmida	Paradoxosomatidae	Asiomorpha	*Asiomorpha coarctata*	KU721885.1	15,644
		Polydesmidae	Epanerchodus	*Epanerchodus koreanus*	MT898420.1	15,581
		Xystodesmidae	Appalachioria	*Appalachioria falcifera*	JX437063.1	15,828
	Spirobolida	Spirobolidae	Narceus	*Narceus annularus*	AY055727.1	14,868
			Spirobolus	*Spirobolus bungii*	MT767838.1	14,879
		Pachybolidae	Litostrophus	*Litostrophus scaber*	OR139892.1	15,081
	Spirostreptida	Odontopygidae	Chaleponcus	*Chaleponcus netus*	MT394513.1	15,093
		Spirostreptidae	Archispirostreptus	*Archispirostreptus gigas*	MT394525.1	15,117
			Macrolenostreptus	*Macrolenostreptus orestes*	MT394512.1	15,367
			Pseudotibiozus	*Pseudotibiozus cerasopus*	MT394506.1	15,121
			Prionopetalum	*Prionopetalum kraepelini*	MT394524.1	15,114
			Tropostreptus	*Tropostreptus austerus*	MT394523.1	15,261
				*Tropostreptus droides*	MT394522.1	15,172
				*Tropostreptus hamatus*	MT394521.1	15,156
				*Tropostreptus kipunji*	MT394511.1	15,170
				*Tropostreptus microcephalus*	MT394516.1	15,169
				*Tropostreptus severus*	MT394517.1	15,209
				*Tropostreptus sigmatospinus*	MT394526.1	15,176
Chilopoda	Lithobiomorpha	Henicopidae	Cermatobius	*Cermatobius longicornis*	KC155628.1	16,833

**Table 2:** Gene annotations of the complete mitogenomes of *L. scaber*.

Gene Name	Location		Length (bp)	Intergenic	Codon		Stand
	From	To			Start	Stop	
trnK	1	56	56				J
COX2	66	744	679	9	ATG	T	J
COX1	745	2269	1525		ATA	T	J
trnW	2287	2347	61	17			J
ND2	2348	3340	993		ATC	TAA	J
trnM	3341	3406	66				J
trnI	3407	3474	68				J
D-loop	3475	3933	459				/
trnL1	3934	3995	62				N
trnP	4007	4067	61	11			N
ND4L	4068	4352	285		TGA	TAG	N
ND4	4346	5683	1338	−6	ATG	TAA	N
trnQ	5686	5751	66	2			N
trnC	5834	5902	69	82			N
rrnS	6020	6776	757	117			N
trnV	6777	6830	54				N
rrnL	7000	8030	1031	169			N
trnL2	8097	8161	65	66			N
ND1	8162	9087	926		ATT	TA	N
trnH	9088	9152	65				N
ND5	9192	10,854	1663	39	ATA	T	N
trnF	10,855	10,912	58				N
trnY	10,913	10,974	62				N
trnT	10,983	11,045	63	8			J
trnS2	11,048	11,114	67	2			J
CYTB	11,118	12,219	1102	3	ATT	T	J
ND6	12,238	12,676	439	18	ATA	T	J
trnE	12,695	12,757	63	18			J
trnS1	12,758	12,817	60				J
trnN	12,818	12,884	67				J
trnR	12,886	12,948	63	1			J
trnA	12,949	13,007	59				J
ND3	13,008	13,355	348		ATT	TAG	J
trnG	13,356	13,417	62				J
COX3	13,418	14,198	781		ATG	T	J
ATP6	14,199	14,871	673		ATG	T	J
ATP8	14,865	15,020	156	−6	ATT	TAA	J
trnD	15,021	15,081	61				J

## Data Availability

DNA sequences: GenBank accession number OR139892.1 for *Litostrophus scaber*.

## Supplementary Materials

The following supporting information can be downloaded at: https://www.mdpi.com/article/10.3390/genes15020254/s1, Table S1: The best-fit models obtained using ModelFinder for ML and BI trees.
