# Supplementary material for: The First Complete Mitochondrial Genome of the Genus Litostrophus: Insights into the Rearrangement and Evolution of Mitochondrial Genomes in Diplopoda
Source: Genes (Basel). 2024 Feb 18;15(2):254. doi: 10.3390/genes15020254 (PMC10888367; doi:10.3390/genes15020254)
Supplement: Supplementary file 1 [file genes-15-00254-s001.zip › genes-2830115-supplementary.pdf]

Table S1: The best-fit models obtained using ModelFinder for ML and BI trees.

Tree	Subset partitions	Best-fit model
ML	CYTB, ATP6, COX2, ND3	GTR+F+I+G4
	ATP8, trnS1	HKY+F+I+G4
	COX1, COX2	TIM2+F+I+I+R4
	ND1, ND4L, ND4, ND5, trnT, trnC	GTR+F+R5
	ND2, ND6, trnW, trnP, trnI	TIM3+F+I+G4
	trnY, trnV, trnS2, trnR, trnQ, trnN, trnM, trnL1, trnH, trnG, trnF, trnE, trnD, trnA, rrnS, rrnL	TVM+F+R4
	trnL2, trnK	TVM+F+G4
BI	CYTB, ATP6, COX2, ND3	GTR+F+I+G4
	ATP8, trnS1	HKY+F+I+G4
	COX1, COX2	GTR+F+I+G4
	ND1, ND4L, ND4, ND5, trnT, trnC	GTR+F+I+G4
	ND2, ND6, trnW, trnP, trnI	GTR+F+I+G4
	trnY, trnV, trnS2, trnR, trnQ, trnN, trnM, trnL1, trnH, trnG, trnF, trnE, trnD, trnA, rrnS, rrnL	GTR+F+G4
	trnL2, trnK	GTR+F+G4
